# Quantifying Breast Cancer-Driven Fiber Alignment and Collagen Deposition in Primary Human Breast Tissue

**DOI:** 10.3389/fbioe.2021.618448

**Published:** 2021-03-15

**Authors:** Rakesh Gurrala, C. Ethan Byrne, Loren M. Brown, Rafael Felix P. Tiongco, Margarite D. Matossian, Jonathan J. Savoie, Bridgette M. Collins-Burow, Matthew E. Burow, Elizabeth C. Martin, Frank H. Lau

**Affiliations:** ^1^Department of Surgery, Louisiana State University Health Sciences Center New Orleans, New Orleans, LA, United States; ^2^School of Medicine, Tulane University, New Orleans, LA, United States; ^3^Department of Biological and Agricultural Engineering, Louisiana State University, Baton Rouge, LA, United States; ^4^Section of Hematology and Medical Oncology, School of Medicine, Tulane University, New Orleans, LA, United States; ^5^Department of Pharmacology, School of Medicine, Tulane University, New Orleans, LA, United States

**Keywords:** breast cancer, extracellular matrix, fiber alignment, collagen content, second harmonic generation, scanning electron microscopy

## Abstract

Solid tumor progression is significantly influenced by interactions between cancer cells and the surrounding extracellular matrix (ECM). Specifically, the cancer cell-driven changes to ECM fiber alignment and collagen deposition impact tumor growth and metastasis. Current methods of quantifying these processes are incomplete, require simple or artificial matrixes, rely on uncommon imaging techniques, preclude the use of biological and technical replicates, require destruction of the tissue, or are prone to segmentation errors. We present a set of methodological solutions to these shortcomings that were developed to quantify these processes in cultured, *ex vivo* human breast tissue under the influence of breast cancer cells and allow for the study of ECM in primary breast tumors. Herein, we describe a method of quantifying fiber alignment that can analyze complex native ECM from scanning electron micrographs that does not preclude the use of replicates and a high-throughput mechanism of quantifying collagen content that is non-destructive. The use of these methods accurately recapitulated cancer cell-driven changes in fiber alignment and collagen deposition observed by visual inspection. Additionally, these methods successfully identified increased fiber alignment in primary human breast tumors when compared to human breast tissue and increased collagen deposition in lobular breast cancer when compared to ductal breast cancer. The successful quantification of fiber alignment and collagen deposition using these methods encourages their use for future studies of ECM dysregulation in human solid tumors.

## Introduction

The extracellular matrix (ECM) is a complex network of macromolecules which provides biochemical and biomechanical signals that govern cell behavior (Walker et al., 2018). Peritumoral dysregulation of the ECM, specifically fiber linearization and altered collagen deposition, is central in solid tumorigenesis and metastasis (Taufalele et al., 2019). Fiber alignment signals cancer cell migration, intravasation, and epithelial–mesenchymal transition (Provenzano et al., 2006; Han et al., 2016; Lee et al., 2017; Zanotelli et al., 2018). Altered and increased collagen deposition increases the ECM’s stiffness, which promotes cancer progression and increased resistance to chemotherapeutics (Taufalele et al., 2019). Reproducibly and accurately quantifying these processes is key to understanding and potentially halting this remodeling. However, current methods of quantifying changes in ECM fiber alignment and collagen deposition are inadequate for human solid tumors. The three primary gaps are: (1) a lack of methods to visualize and quantify fiber alignment changes in common ECM images such as those captured by scanning electron microscopy (SEM); (2) the absence of generalized statistical methods to combine and analyze fiber alignment frequency distributions across multiple samples; and (3) a lack of methods to reliably and accurately quantify changes in the collagen content of light microscopy images. Together, these gaps inhibit the study of ECM in human solid tumors.

Fiber alignment quantification requires imaging of the ECM, computational identification of fibers in micrographs, measurement of fiber orientations followed by calculation of fiber intersection angles. With current techniques’ shortcomings, fiber imaging for quantitative analysis requires second harmonic generation (SHG) microscopy to provide high-resolution data regarding collagen structure and alignment (Keikhosravi et al., 2014). However, SHG microscopy is cost-prohibitive and not widely available (Chen et al., 2012; Keikhosravi et al., 2014). This reliance on SHG is due to limitations in the computational methods developed to study fiber alignment thus far. Specifically, the collagen analysis software CurveAlign and its companion program CT-FIRE have only been reported in the study of non-physiologic ECMs, such as artificial collagen gels and only using SHG micrographs (Provenzano et al., 2006; Majeed et al., 2017). When applied to more commonly available imaging modalities such as SEM, the reported algorithms fail to reliably differentiate between fibers from background. These shortcomings are exacerbated when applied to more complex ECM, such as that found in human breast cancer (Bredfeldt et al., 2014a,b). Conklin et al. (2018) utilized CT-FIRE to study SHG micrographs of human breast cancer biopsies, but some fibers were over-or under-segmented.

The collection of fiber orientations for a given matrix constitutes a distribution, which can be represented as a histogram. For simple, artificial matrices such as those produced using a single collagen, this distribution is normally (Gaussian) distributed. However, in native human ECM, the distributions are non-parametric and typically have multiple, non-Gaussian peaks. Previous studies reported single-number metrics, such as standard deviation (SD), alignment index (AI), and orientation index (OI) (Sun et al., 2015; Taufalele et al., 2019), as characterizations of these distributions. These single-number metrics were designed for use with simple single collagen matrices and lack accuracy when applied to complex ECM. For native human ECM, these simple metrics are insufficient. Furthermore, we are unaware of any published methods which allow for (1) combining distributions from technical and biological replicates and (2) statistically determining if distributions from different experimental conditions significantly vary. This is evidenced by fiber alignment studies reporting a single histogram for each experimental group without reporting significance (Grossman et al., 2016).

The second component to ECM remodeling is altered collagen deposition. While non-destructive histological stains can be used to qualitatively evaluate collagen deposition, semi-quantitative methods have historically relied on destructive methods such as Western blots. Previously reported image-based methods were time-consuming and inaccurate as they required manually segmenting areas of interest to separate collagen from image background (Schipke et al., 2017; Rieppo et al., 2019). Furthermore, in human ECM, multiple biological components can stain similarly to collagen, making color-dependent thresholding methods prone to inaccuracies.

Herein, we present solutions for these major methodological challenges. First, we demonstrate that segmenting ECM fibers within a SEM micrograph using a trainable segmentation tool prior to DiameterJ analysis allows accurate fiber detection from complex fiber networks. Second, we report a method for normalizing multiple collagen orientation frequency distributions to allow biological and technical replicates to be combined and compared for statistically different changes. Additionally, we describe a high-throughput machine-learning-based technique to accurately quantify collagen content from histological sections. Together, these methods will allow for robust quantitative analysis and reporting of ECM remodeling data, enabling the accurate study of human solid tumors.

## Materials and Methods

All human tissue samples were collected in adherence to protocols #8759 and # 9189, as approved by the IRB Office of Louisiana State University Health Sciences Center (LSUHSC).

### ECM Generation

**FIGURE 1:**
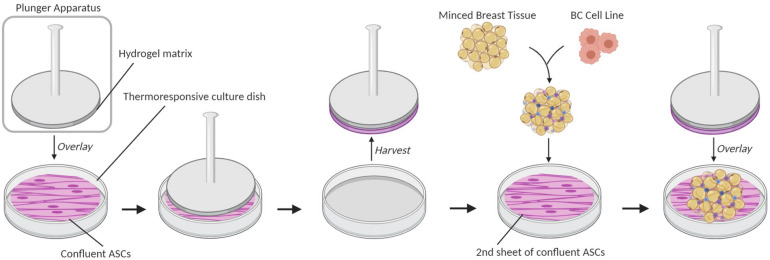
Breast cancer microphysiological system (BC-MPS) generation. Schematic of BC-MPS generation showing transfer of confluent ASC cell sheet from UpCell^TM^ (Nunc) thermoresponsive dishes onto a second sheet of confluent ASCs grown on a standard plastic tissue culture dish. Sandwiched between the two ASC sheets is minced primary breast tissue seeded with breast cancer cell lines.

To acquire images of ECM fibers, breast cancer microphysiological systems (BC-MPSs) were generated by culturing primary breast tissue between two sheets of adipose-derived stromal cells (ASCs); this was a modification of a previously described method for culturing adipose tissues (Lau et al., 2018; Scahill et al., 2018). Briefly, six-well culture plates were seeded with ASCs (Figure 1). Base layer cell sheets were cultured on tissue-culture plastic dishes (Corning), whereas top layer sheets were cultured on UpCell^TM^ (Nunc) thermoresponsive dishes. A 7.5% gelatin solution was solidified on a plunger apparatus and placed upon upper layer cell sheets at room temperature for 1.5 h to adhere the plunger to the cell sheet. The upper layer cell sheets were then released from the UpCell^TM^ dishes by treatment in an ice water bath for 1.5 h. Breast tissue was acquired from patients undergoing elective surgery, washed with PBS, minced, and combined with Dulbecco’s modified Eagle’s medium (DMEM). MDA-MB-231 breast cancer cells were seeded into the minced breast tissue. 300 μL of the BC cell-breast tissue mixture was aliquoted onto bottom layer cell sheets. Upper layer cell sheets were transferred on top of the aliquoted BC cell-breast tissue mixture using the plunger apparatus. Warmed culture media were added to each well prior to a 30-min incubation at 37°C to release the upper cell sheet from the plunger apparatus. Samples were grown for 14 days prior to tissue decellularization.

### Cell Culture

Adipose-derived stromal cells were isolated from primary human breast tissue using a previously described methodology (Lau et al., 2018). ASCs were cultured in DMEM supplemented with 1% Pen/Strep antibiotics (Gibco, Dublin, Ireland) and 10% FBS (Atlanta Biologicals, Georgia). MDA-MB-231 cells were cultured in DMEM supplemented with 10% Serum (HyClone Cosmic Calf, state), 50 ng/mL insulin (Sigma–Aldrich, St. Louis, MO, United States), and 1% MEMAA, NEAA, sodium pyruvate, and antibiotic-antimycotic (Gibco, Waltham, MA, United States). All cells were maintained in 5% CO2 at 37°C.

### Primary Breast Tumor

The metaplastic breast tumor model, known as TU-BcX-4IC, was obtained from a mastectomy of a 57-year-old white female with metaplastic breast carcinoma unresponsive to neoadjuvant adriamycin/cyclophosphamide therapy as previously described (Chang et al., 2020). The tumor was obtained from the surgical specimen just after mastectomy. The mastectomy specimen was confirmed pathologically as a TNBC subtype. The tumor was obtained in collaboration with the Louisiana Cancer Research Center Biospecimen Core, which obtains tumor specimens from local hospitals.

### Tissue Decellularization

Breast cancer microphysiological system samples grown for 14 days and the metaplastic breast tumor specimen were decellularized through a modified previously described protocol (Pashos et al., 2017). Samples were collected from six-well dishes using a cell scraper, then transferred to cryovials, and stored in liquid nitrogen until ready to decellularize. Samples were thawed at room temperature and then washed with PBS prior to decellularization. Samples were then incubated on a shaker at 37°C, 70 r/min with the following reagents: diH_2_O for 2 h, Triton-X for 48 h (Sigma–Aldrich, St. Louis, MO, United States) diH_2_O for 2 h, sodium deoxycholate solution (Amresco, Solon, OH, United States) for 48 h, diH_2_O for 2 h, sodium chloride for 2 h, and diH_2_O for 2 h. Samples were stored at 4°C in a PBS solution containing 5x antibiotic/antimycotic until use.

### Scanning Electron Microscopy

Decellularized samples were fixed in formalin-acetic acid-alcohol (FAA) overnight. After fixation, samples were dehydrated using graded ethanol concentrations of 50, 70, and 90%, one time for each and three times in 100% for 30–60 min each. Following the final 100% ethanol wash, samples were further dried by Critical Point Drying using dry siphoned liquid CO_2_. Samples were then spray coated and imaged at 25k magnification using FEI Quanta 3D FEG FIB/SEM. Samples exhibited little heterogeneity and representative images were used for analysis.

### SEM Image Analysis

Six SEM micrographs of the metaplastic breast tumor specimen and three SEM micrographs of 14 decellularized BC-MPS samples and 14 decellularized BC-MPS samples seeded with MDA-MB-231 cells were analyzed using modified previously described methodology (Hotaling et al., 2015). Briefly, SEM micrographs of decellularized ECM were contrast adjusted in ImageJ to move the lowest grayscale pixel intensity to a true black value. Contrast-adjusted micrographs were then segmented using the WEKA Trainable Segmentation (WTS) tool in ImageJ (Arganda-Carreras et al., 2017). The WTS tool was trained using prior images of tumor fibers to effectively identify fibers and fiber edges. After segmentation, remaining noise was removed with ImageJ’s despeckle command. The micrographs were converted to an 8-bit image for processing with the DiameterJ plugin for ImageJ. Fiber orientations were calculated using the OrientationJ plugin within ImageJ. Briefly, an axial thinning algorithm was used to generate a centerline for each fiber in each image. A Fourier gradient with a Gaussian window of 7 pixels was applied to each center line. The angle, relative to an arbitrary line determined by the OrientationJ, at which each center line points was recorded and displayed in a frequency histogram with angles ranging from −90° to +89°. This method is hereon referred to as the WEKA-DiameterJ (WEKA-DJ) method. Additionally, each SEM micrograph was analyzed using CT-FIRE and subsequently CurveAlign software following the manufacturer’s instructions. CT-FIRE is used to extract individual fibers from a micrograph. CurveAlign then uses the extracted fiber data from CT-FIRE to compute fiber orientations and alignment (Liu et al., 2017). Oversegmentation incidence was calculated by manually counting oversegmentation errors in five CT-FIRE Maps and corresponding Axial Thinning Maps generated from SEM micrographs of distinct ECMs. Segmented fibers were considered erroneous if they were found in pores where no fibers were visible or if multiple fibers were segmented along the width of one visible fiber.

### Fiber Orientation Histogram Normalization

**FIGURE 2:**
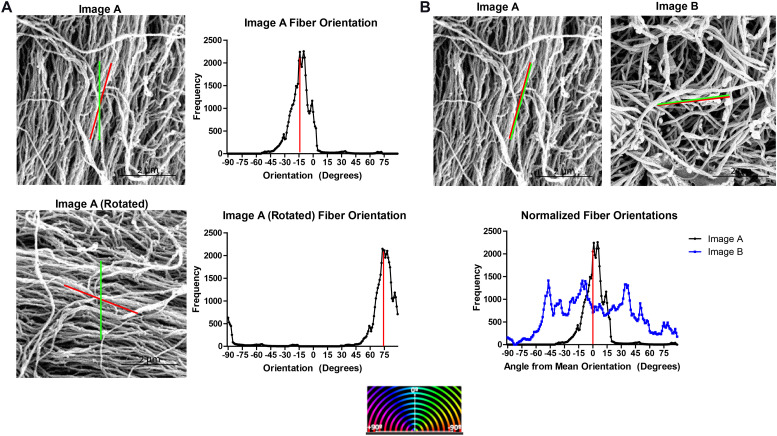
Normalizing ECM fiber orientation distributions allows comparison of distinct samples and combination of replicates. **(A)** Fiber orientation distribution output by DiameterJ for a SEM micrograph of decellularized ECM (Image A) and the same image rotated 90° for which the mean fiber orientation (red line) and orientation set as 0° by DiameterJ (green line) are superimposed. Red stripes indicate the distribution’s mean. **(B)** Superimposed normalized fiber orientation distributions for Image A and a separate SEM micrograph, Image B, generated by subtracting the mean fiber orientation from all angle orientations, shifting the distributions’ means to 0°.

Fiber orientation distributions analyzed from different SEM micrographs cannot be directly compared since the orientation assigned to each fiber is an angle relative to an arbitrary line set by the DiameterJ software. Figure 2A shows the orientation histograms generated for a SEM micrograph, Image A, and for the same SEM micrograph rotated 90° clockwise, Image A Rotated. Despite analyzing the same SEM micrograph, two different orientation distributions are generated using the DiameterJ plugin.

To normalize the fiber orientation distributions, the mean fiber orientation was calculated for each SEM micrograph and subtracted from all fiber orientations generating orientations relative to the mean fiber orientation and shifting the mean of each distribution to the center of the histogram. Values that have been shifted outside of the −90° to 89° range of orientations are then transferred back in range by adding ±180°. Normalizing the fiber orientation distributions allows multiple distributions to be superimposed on each other for comparison, such as Image A and Image B (Figure 2B).

Distributions with normalized data from technical replicates (*n* = 3) and biological replicates (*n* = 14) were averaged together by summing together frequencies at each orientation relative to the mean fiber orientation and dividing by *n*.

### Orientation Index

Orientation indices were generated from the fiber orientation distributions by implementing a modified previously described method in Microsoft Excel (Taufalele et al., 2019). Briefly, the OI, S, is defined by:

$$S=2\,<f\,\left(\frac{1}{2}\,\cos\left(2\,\alpha \right)+1\right)>-1$$

where *f* represents the normalized frequency, α represents the angle between an individual fiber and the mean fiber orientation for that distribution, and $<f\,\left(\frac{1}{2}\,\cos\left(2\,\alpha \right)+1\right)>$ represents the averaged square cosine of all α per image. An OI of 0 represents a perfectly random distribution, whereas an OI of 1 represents a perfectly aligned distribution.

### Collagen Quantification

Four samples of BC-MPS grown for 14 days with and without MDA-MB-231 cells were fixed in 10% formalin at 4°C overnight, then paraffin embedded, and sectioned at 5 μm using a Leica RM 2235 microtome. Four 5 × 5 μm frozen sections of lobular carcinoma and six sections of ductal carcinoma were purchased form Origene (Rockville, MD, United States). Masson’s trichrome staining for collagen was accomplished on all tissue sections using a standard protocol and imaged using a Mere PathScan 5 slides canner (Campbell et al., 2014).

A random forest machine learning method for identifying pixels as collagen positive, collagen negative, or image background was trained using the QuPath Pixel Classifier for five Masson’s Trichrome stains of BC-MPS and frozen sections of ductal and lobular carcinoma (Bankhead et al., 2017). Collagen percentage for each specimen was calculated by dividing collagen positive pixels determined by the pixel classifier by total pixels remaining in the image after background removal.

### Statistical Analysis

Mann–Whitney *U* tests were performed on averaged normalized frequency distributions using Microsoft Excel (Microsoft, Redmond, WA, United States). Two-way ANOVA followed by Tukey’s multiple comparison test was performed on averaged OI data and collagen percentage data using GraphPad Prism 5 (GraphPad Software, La Jolla, CA, United States).

## Results

### Accurate and Rapid Fiber Orientation Detection With Combined WEKA-DiameterJ Analysis

**FIGURE 3:**
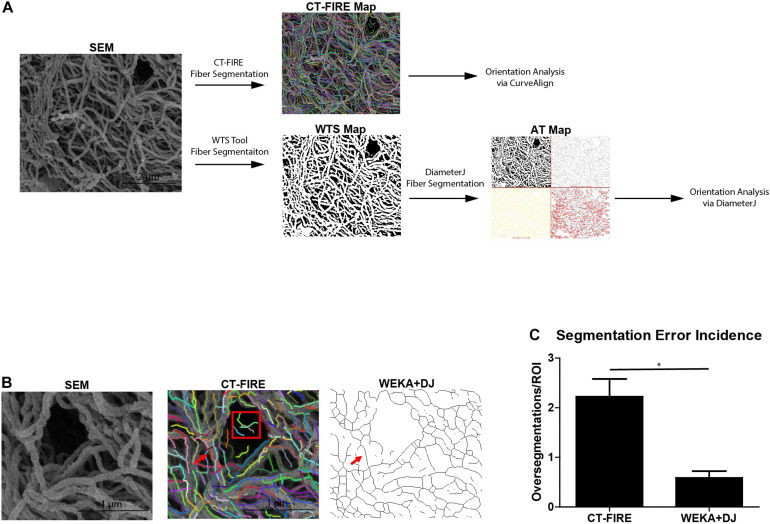
WEKA-DiameterJ method produces more accurate fiber segmentation than CT-FIRE. **(A)** Schematic of workflow for quantifying collagen alignment for a scanning electron micrograph (SEM) using the CT-FIRE method and WEKA-DiameterJ method (WEKA+DJ). Segmented fibers are indicated by colored lines on the CT-FIRE Map, by white pixels on the Weka Segmentation Tool (WTS) Map, and as thin black lines on the Axial Thinning (AT) Map. **(B)** A region of interest within the SEM micrograph and corresponding CT-FIRE Map, and AT Map after segmentation with the WEKA-DJ method. A single foreground fiber (red arrow) within the SEM is oversegmented by CT-FIRE but appropriately segmented by the WEKA-DJ method. Segmentation by CT-FIRE incorrectly segmented fibers within a pore (red box). **(C)** Incidence of oversegmentation (mean + SEM) by CT-FIRE and WEKA-DJ methods (*n* = 5) (*p* = 0.0172).

Prior to determining fiber orientations, fiber alignment quantification software must detect fibers within the SEM micrograph. Figure 3A shows the workflow for analyzing a SEM micrograph by the CT-FIRE and WEKA-DJ method. CT-FIRE is used to extract fibers from a micrograph prior to quantitative fiber analysis by CurveAlign. Fibers segmented by CT-FIRE are indicated by multi-colored lines on a CT-FIRE Map. Alternatively, segmentation of fibers can be done via the WEKA-DJ method in which the WTS Tool is used prior to analysis by DiameterJ. The WTS Tool outputs a probability map in which fibers are indicated in white and pores in black. DiameterJ determines fibers from the WTS Map and generates an axial thinning map illustrating where segmented fibers lie. The WEKA-DJ method allowed for accurate fiber detection without significant rates of fiber oversegmentation. Fiber detection using the CT-FIRE method was prone to oversegmentation errors. The software characterized single fibers as multiple fibers within SEM micrographs and incorrectly identified fibers in large pores (Figure 3B). Fiber segmentation using CT-FIRE produced 23.90 ± 8.83 (mean ± SEM) oversegmentation errors per 6 μm × 6 μm SEM micrograph, relative to 5.91 ± 2.09 using the WEKA-DJ method (*p* = 0.017) (Figure 3C).

### Normalized Orientation Distributions Can Be Used to Quantify Differences in ECM Fiber Alignment

**FIGURE 4:**
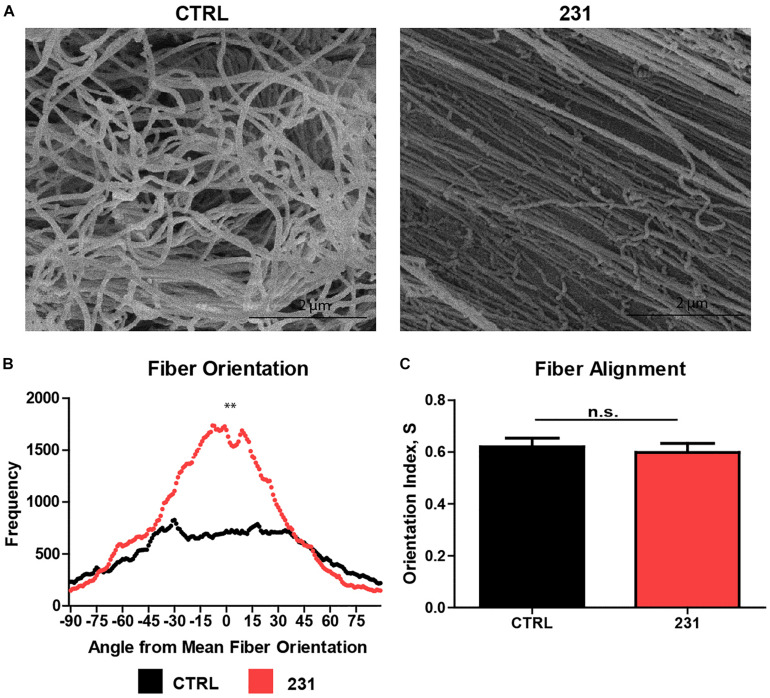
Normalized orientation distributions identify visible differences in ECM fiber alignment. **(A)** SEM micrographs of decellularized ECM generated using breast tissue alone (CTRL) and seeded with MDA-MB-231 cancer cell lines (231). **(B)** Normalized fiber orientation distributions for CTRL and 231 ECM (*n* = 14) (*p* = 0.000114). **(C)** Orientation index (mean + SEM) for CTRL and 231 ECM (*n* = 14) (*p* = 0.6520).

Normalizing fiber orientation data from DiameterJ using the mean fiber orientation method allows data from technical replicates and between experimental groups to be combined and compared, respectively. Collating data from multiple replicates is a fundamental tenet of both experimental biology and statistical analysis of differences arising under varying experimental conditions. SEM micrographs of ECM show a greater degree of fiber alignment in samples treated with MDA-MB-231 cancer cells than in untreated ECM (Figure 4A). Mann–Whitney *U* tests on the normalized orientation distributions accurately identified significant differences in fiber alignment between ECM which was untreated and ECM treated with the breast cancer cell line (*p* = 0.0001) (Figure 4B). In contrast, the OI for control ECM was 0.621 ± 0.047 (mean ± SEM) vs. 0.599 ± 0.040 in cancer-treated ECM (*p* = 0.6520) (Figure 4C).

### Pixel Classification Allows Quantitative Histochemical Analysis of Collagen Content

**FIGURE 5:**
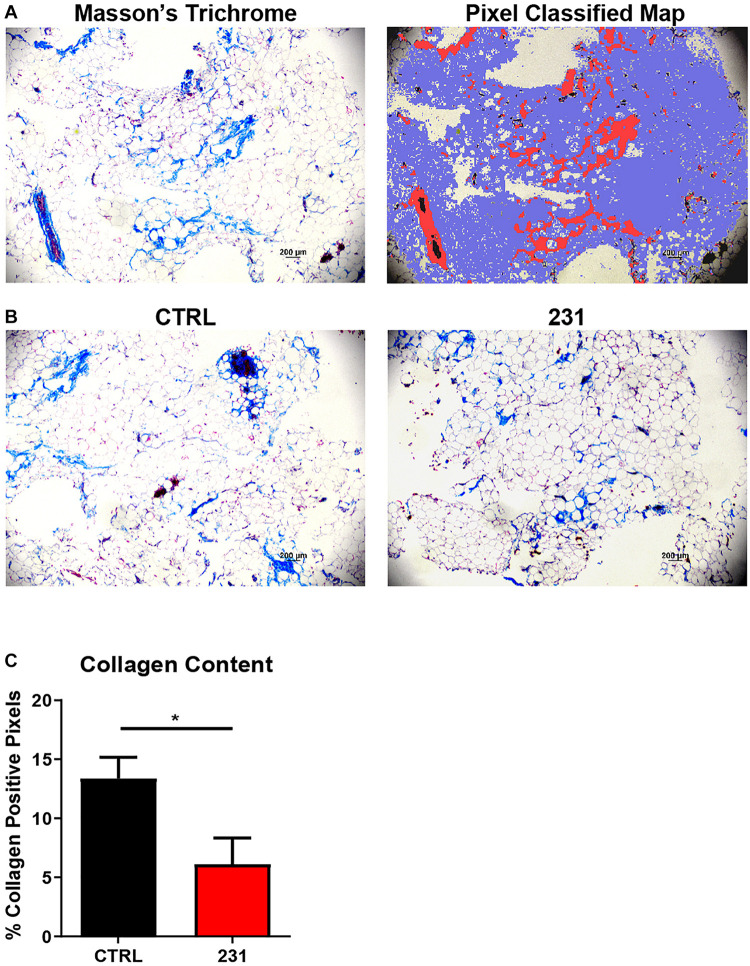
Pixel classification allows quantitative histochemical analysis of collagen content. **(A)** Masson’s Trichrome stain of BC-MPS and corresponding probability map generated by pixel classification, in which red pixels indicate collagen deposition, blue pixels indicate areas with no collagen, and white pixels indicate background. **(B)** Masson’s Trichrome stains of BC-MPS alone (CTRL) and BC-MPS under the influence of MDA-MB-231 cancer cells (231). **(C)** Collagen content (mean + SEM) of BC-MPS alone (CTRL) (*n* = 4) and under the influence of MDA-MB-231 cells (231) (*n* = 6) (*p* = 0.0430).

Quantitative histochemical analysis of collagen content was performed on BC-MPS using a random tree-based machine learning algorithm. The trained pixel classifier accurately identified pixels within Masson’s trichrome stains as collagen, not collagen, or background when analyzing BC-MPS (Figure 5A). Masson’s trichrome stains of BC-MPS with MDA-MB-231 cells exhibited less collagen content than BC-MPS alone (Figure 5B). The trained pixel classifier determined that on average 13.39 ± 3.56 and 6.12 ± 4.42% of pixels were collagen positive for BC-MPS and BC-MPS with the breast cancer cell line, respectively (*p* = 0.0430) (Figure 5C).

### Normalized Orientation Distributions and Pixel Classification Allow Quantification of ECM Dysregulation in Primary Breast Tumors

**FIGURE 6:**
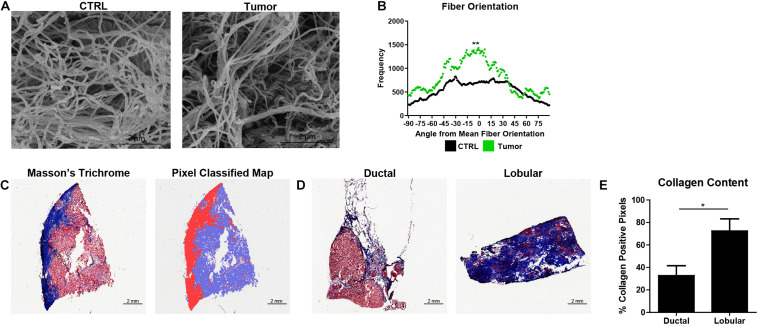
Normalized orientation distributions and pixel classification allow quantification of ECM dysregulation in primary breast tumors. **(A)** SEM micrographs of decellularized ECM from human breast tissue (CTRL) and decellularized ECM from a primary human breast tumor. **(B)** Normalized fiber orientation distributions for the human breast tissue (CTRL) (*n* = 14) and breast tumor ECM (*n* = 1) (*p* = 0.003221). **(C)** Masson’s Trichrome stain of frozen breast section and corresponding probability map generated by pixel classification, in which red pixels indicate collagen deposition, blue pixels indicate areas with no collagen, and white pixels indicate background. **(D)** Masson’s Trichrome stains of lobular carcinoma and ductal carcinoma. **(E)** Collagen content (mean + SEM) of lobular carcinoma (*n* = 4) and ductal carcinoma (*n* = 6) (*p* = 0.026779). *Indicates *p* < 0.05 and ** indicates *p* < 0.005.

Fiber orientation analysis and quantitative histochemical analysis of collagen content from primary human breast tumors were performed utilizing the WEKA-DJ method and pixel classifier, respectively. SEM micrographs of ECM show a greater degree of fiber alignment in tumors than in human breast tissue cultured in the BC-MPS (Figure 6A). Mann–Whitney *U* tests on the normalized orientation distributions accurately identified the visually observed differences in fiber alignment (*p* = 0.003221) (Figure 6B). The trained pixel classifier accurately identified pixels within Masson’s trichrome stains as collagen, not collagen, or background in the sections of primary breast tumors (Figure 6C). Masson’s trichrome stains of lobular carcinoma exhibited more collagen content than ductal carcinoma (Figure 6D). The trained pixel classifier determined that on average 73.40 ± 8.87 and 32.69 ± 10.87% of pixels were collagen positive for lobular carcinoma and ductal carcinoma, respectively (*p* = 0.027) (Figure 6E).

## Discussion

Given the importance of ECM remodeling in cancer progression and metastasis, accurate and accessible methods of quantifying changes in fiber alignment and collagen deposition in human samples are needed (Walker et al., 2018). Software tools have been developed to measure such changes from micrographs of ECM. CurveAlign and CT-FIRE have both been successfully applied to study simplified artificial and murine collagen matrices. However, both programs have difficulty identifying (1) intact fibers from a dense fiber network, (2) curvy fibers, (3) fibers which have large variations in inter-fiber brightness, and (4) dark fibers adjacent to brighter ones (Drifka et al., 2015; Walsh et al., 2015; Liu et al., 2017). Previous studies utilizing CurveAlign and CT-FIRE have relied on SHG microscopy, which provides high contrast between collagen fibers and their background, reducing the software’s fiber detection errors (Chen et al., 2012). Other imaging modalities such as SEM do not share the same sensitivity to fibrillar collagen, precluding the use of a commonly available and critical imaging modality from the study of ECM (Bredfeldt et al., 2014b). Modern SHG instrumentation requires a femtosecond laser and a laser scanning optical microscope making the imaging modality cost prohibitive. The fiber detection challenges using contemporary software are exacerbated when human tumors were imaged; CT-FIRE over- and under-segmented fibers despite the use of SHG microscopy (Conklin et al., 2018).

In this study, we demonstrated that segmentation of SEM micrographs using a machine learning tool prior to orientation analysis addressed these challenges and accurately identified fiber alignment in native, human breast ECM. Prior to fiber orientation analysis with DiameterJ, SEM micrographs were segmented using the WTS Tool. The WTS Tool is a pixel-based segmentation software which is built upon the robust machine learning and data mining platform, Waikato Environment for Knowledge Analysis (WEKA) (Arganda-Carreras et al., 2017). The sophistication and adeptness of the WEKA platform allow for accurate fiber determination when trained with SEM micrographs. In contrast, CurveAlign and CT-FIRE rely on de-noising the SEM micrograph using a curvelet transform and a variety of post-processing algorithms to identify fibers. CT-FIRE uses the “FIRE” algorithm which contains steps for boundary detection, fiber center point identification, and short fiber removal. In CurveAlign, individual fibers are not extracted, rather the curvelets from a curvelet transform are used to represent the edges of fiber segments without additional steps to detect fiber edges, hence fiber extraction is done by CT-FIRE prior to analysis by CurveAlign (Liu et al., 2017). Unlike the WTS tool which can be trained for any image type, the curvelet-based methods require high-resolution images of collagen fibers and are prone to inaccuracies. Thus, utilization of the WTS tool allows accurate fiber alignment analysis from lower resolution imaging methods such as ECM.

However, the use of the WTS tool requires the subsequent use of DiameterJ. DiameterJ outputs a frequency distribution of fiber orientations instead of a single-number metric like CurveAlign’s alignment coefficient. A method of comparing alignment using frequency distributions across samples was necessary. Previous methods of assigning single-number metrics such as OI to DiameterJ’s frequency distributions have been described. Like other single-number metrics, OI is a valuable tool only when the distribution is Gaussian. Single-number metrics cannot represent the complexity of real ECM distributions, which typically have multiple, non-Gaussian peaks. The method described here allows two non-parametric frequency distributions to be compared. This opens the door to the use of technical and biological replicates. This is critical when working with complex, primary human ECM which has much greater inter-image fiber variation compared to artificial, single collagen matrices used in prior studies. Here, we showed that OI would have incorrectly affirmed a lack of difference in alignment among samples which contain visible differences in fiber alignment. Furthermore, MDA-MB-231 cells have been previously shown to induce ECM fiber alignment in a variety of 3D ECM models (Riching et al., 2014). Currently, studies establish significant changes in cancer ECM utilizing genomic and proteomic findings (Assadian et al., 2012; Álvarez-Garcia et al., 2019). Although these findings indicate molecular changes in ECM, they do not shed light on the physiological significance of cancer related ECM re-organization. The ability to quantify ECM reorganization from imaging and compare across samples allows for future study into this area.

Similar gaps in quantifying collagen content in ECM exist. Current easily-accessible and accurate methods of quantifying collagen content rely on destructive methods such as HPLC or hydroxyproline assays which fail to provide information about the spatial distribution of collagen content within a specimen (Qiu et al., 2014). Collagen content was quantified for sections stained with Picrosirius red, but the method required a polarized light microscope which is cost-prohibitive (Wegner et al., 2017). A semiautomated method of histochemical quantification of collagen content was described using a threshold analysis but was imprecise as it included non-collagen structures as collagen and failed to detect smaller collagen deposits (Schipke et al., 2017). Other methods of quantifying collagen from histochemical stains rely on manual selection of collagen which is prone to subjective errors. Training a pixel classifier using a random forest decision making algorithm makes it possible to incorporate vastly more information into identifying collagen during image analysis than is possible with a simple threshold-based system, thus enabling accurate identification of collagen content (Bankhead et al., 2017). We demonstrated the use of QuPath’s trainable pixel classifier as an adept way of determining collagen from Masson’s Trichrome stains of complex microphysiological models.

The methods to quantify ECM dysregulation described here were successfully applied to primary human breast tumors. Fiber alignment quantification using the WEKA-DJ method determined that ECM was significantly more aligned in tumors than in native human breast tissue. This is expected as tumors have been shown to induce fiber linearization within the ECM. As this is a critical mechanism for tumor migration and progression, further examination of ECM fiber alignment using this method may provide insight into tumor behavior. Additionally, the pixel classifier successfully identified significant differences in collagen content between ductal and lobular carcinomas from histochemical sections. Identification of histological differences between lobular and ductal breast cancer types has clinical utility, as these two cancers are differentiated via examination of histological sections. Previously, no differences in collagen content between ductal and lobular tumors were reported with the use of SHG microscopy despite differences in collagen associated gene expression between the cancer subtypes. This discrepancy illustrates increased adeptness of these methods to analyze ECM.

The use of these methods can also be utilized to analyze cell-based therapies used to treat injured or degenerated tissues. Zeugolis et al. described macromolecular crowding (MMC), a biophysiological approach to cell-based therapy in which inert polydispersed macromolecules are added to culture media to create ECM-rich tissue equivalents. MMC induced a plethora of changes to the ECM, such as increased deposition of numerous collagenous proteins, basement membrane proteins, and stroma remodeling enzymes (Satyam et al., 2014). Further evaluation of MMC using the WEKA-DJ and pixel classification methods may provide insight into the structural remodeling the ECM undergoes with MMC.

In cancers, remodeled ECM protects the tumor by impeding drug diffusion and activation of drug resistance pathways by hypoxia. Hence, we plan to examine the role of altered ECM fiber alignment and collagen deposition in drug resistance in future studies utilizing the methods described in this study.

## Conclusion

Quantitative analysis of the ECM is becoming more prominent as the ECM is increasingly recognized as an important regulator in a variety of diseases including breast cancer. Contemporary tools to quantify two key mechanisms of cancer-associated ECM dysregulation, fiber alignment and collagen deposition, are inaccurate or cost prohibitive. The use of the WEKA-DJ and pixel quantification methods to quantify these processes is accurate, affordable, and adaptable. Furthermore, these methods have successfully been used to quantify fiber alignment and collagen deposition in human solid tumors and thus will allow robust study of the role of ECM dysregulation in cancer.

## Data Availability Statement

The raw data supporting the conclusions of this article will be made available by the authors, without undue reservation.

## Author Contributions

RG conceived of the presented analytical methods. RG, CB, and RT performed the computational analysis. LB, EM, and FL verified the analytical methods. RG, LB, and RT generated the BC-MPS model. MM, BC-B, and MB developed the TU-BcX-4IC breast tumor model. CB and JS processed and imaged the EM. FL and EM encouraged RG to investigate fiber alignment and supervised the findings of this work. All authors discussed the results and contributed to the final manuscript.

## Conflict of Interest

The authors declare that the research was conducted in the absence of any commercial or financial relationships that could be construed as a potential conflict of interest.

## Abbreviations

AI: alignment index
BC-MPS: breast cancer microphysiological system
OI: orientation index
SHG: second harmonic generation
WEKA: Waikato Environment for Knowledge Analysis
WEKA-DJ: Weka-DiameterJ
WTS: Weka Trainable Segmentation.
